# The Neuromelanin-related T_2_* Contrast in Postmortem Human Substantia Nigra with 7T MRI

**DOI:** 10.1038/srep32647

**Published:** 2016-09-06

**Authors:** Jae-Hyeok Lee, Sun-Yong Baek, YoungKyu Song, Sujeong Lim, Hansol Lee, Minh Phuong Nguyen, Eun-Joo Kim, Gi Yeong Huh, Se Young Chun, HyungJoon Cho

**Affiliations:** 1 Department of Neurology, Research Institute for Convergence of Biomedical Science and Technology, Pusan National University Yangsan Hospital, Yangsan, South Korea; 2 Department of Anatomy, Pusan National University School of Medicine, Yangsan, South Korea; 3 Department of Biomedical Engineering, Ulsan National Institute of Science and Technology, Ulsan, South Korea; 4 School of Electrical and Computer Engineering, Ulsan National Institute of Science and Technology, Ulsan, South Korea; 5 Department of Neurology, Pusan National University Hospital, Busan, South Korea; 6 Department of Forensic Medicine, Pusan National University School of Medicine, Yangsan, South Korea

## Abstract

High field magnetic resonance imaging (MRI)-based delineation of the substantia nigra (SN) and visualization of its inner cellular organization are promising methods for the evaluation of morphological changes associated with neurodegenerative diseases; however, corresponding MR contrasts must be matched and validated with quantitative histological information. Slices from two postmortem SN samples were imaged with a 7 Tesla (7T) MRI with T_1_ and T_2_* imaging protocols and then stained with Perl’s Prussian blue, Kluver-Barrera, tyrosine hydroxylase, and calbindin immunohistochemistry in a serial manner. The association between T_2_* values and quantitative histology was investigated with a co-registration method that accounts for histology slice preparation. The ventral T_2_* hypointense layers between the SNr and the crus cerebri extended anteriorly to the posterior part of the crus cerebri, which demonstrates the difficulty with an MRI-based delineation of the SN. We found that the paramagnetic hypointense areas within the dorsolateral SN corresponded to clusters of neuromelanin (NM). These NM-rich zones were distinct from the hypointense ventromedial regions with high iron pigments. Nigral T_2_* imaging at 7T can reflect the density of NM-containing neurons as the metal-bound NM macromolecules may decrease T_2_* values and cause hypointense signalling in T_2_* imaging at 7T.

Identifying and characterizing the anatomic architecture of the substantia nigra (SN) has important clinical implications for the evaluation of structural changes associated with neurodegenerative conditions, such as Parkinson’s disease (PD)^1^,^2^,^3^. The SN is subdivided into two histologically distinct regions, the ventral pars reticulata (SNr) and the dorsal pars compacta (SNc). The SNc is composed of neuromelanin (NM)-containing dopaminergic neurons, which are affected early in PD^1^,^2^,^3^.

Numerous attempts have been made to visualize substructure morphology of the SN and to assess the neurodegenerative changes using various magnetic resonance imaging (MRI) signal contrasts^2^,^3^. For example, iron-sensitive MR sequences have great potential to define the boundaries and shape of the SN^2^,^3^,^4^ as the local deposition of iron alters magnetic field inhomogeneities and appears hypointense in T_2_ or T_2_^*^-weighted images (T_2_*WI) due to the shortening of transverse relaxation times^2^,^5^. By taking advantage of region-specific iron content within the SN, the area of lower T_2_^*^-weighted signal intensity is assigned to the SNr based on the histological observation of elevated iron concentration in that region^3^,^5^,^6^. NM-sensitive T_1_-weighted fast spin echo technique in *in vivo* 3T MRI studies allows the visualization of the SNc via hyperintense areas^2^,^3^,^7^,^8^,^9^. NM within the dopaminergic neurons is speculated to generate paramagnetic T_1_-shortening effects on combining with metals, such as iron and copper^2^,^3^,^7^,^10^. While NM-containing dopaminergic neurons are densely distributed in the SNc, they form clusters of cells that penetrate deep into the caudal part of the SNr. Thus, it may not be easy to delineate the boundaries between the SNc and the SNr with the use of NM-sensitive T_1_-weighted MRI alone^3^.

Recently, ultra-high field seven Tesla (7T) MRI has provided detailed morphological information of the SN with improved spatial resolution and T_2_^*^-contrast and has opened up the possibility for more accurate identification of PD pathology^3^,^4^,^5^,^6^,^11^. Specifically, postmortem 7T T_2_^*^WI, in combination with histological correlations and *in vivo* data, can directly depict the pockets of high signal intensity in the dorsal SN corresponding to nigrosome 1, which is known to be the structure most vulnerable to degeneration in PD^1^,^3^,^11^. In PD, the loss of hyperintense nigrosome 1 and the expansion of signal hypointensity were each identified as a result of the loss of dopaminergic neurons and an increase in iron content within nigrosome 1^3^,^5^,^6^,^11^. More recently, 3T susceptibility-weighted imaging (SWI) was used to detect nigrosome 1 by dorsolateral nigral hyperintensity, which is absent in neurodegenerative parkinsonism, including PD^12^,^13^, progressive supranuclear palsy, and multiple system atrophy^14^.

*In vivo* MRI studies have reported an increase in overall iron accumulation without specifying the form of stored iron in the SN^11^. Ferric iron, whose strong paramagnetic properties alter MR signal contrast, is also known to bound to NM and ferritin^4^,^11^. The resulting NM-iron complex is one of the main iron compounds in SN dopaminergic neurons^15^. From the MR perspective, the paramagnetic NM-iron complexes may be detected by T_2_^*^-weighted iron-sensitive MR sequences as well as NM-sensitive T_1_-weighted imaging (T_1_WI), but their efficacy can change with an increasingly magnetic field and requires thorough validation. We hypothesize that NM would significantly contribute to the T_2_*-weighted hypointensity observed in the SN with increasing magnetic field strength. Similarly, the SN regions with a high NM content, such as nigrosome 1, may not appear fully hyperintense in T_2_*WI from the high magnetic field of 7T.

In this study, we investigated the association between postmortem T_2_* imaging at 7T and the histological features in non-specific pathology brain samples to identify the contributions of various histological components to the T_2_* signal loss in the SN. T_2_* maps for an unbiased quantification of distinct physical tissue properties^16^ were generated and directly correlated with quantitative histology, such as densities of NM and iron pigments^17^.

## Methods

This study was approved by the Pusan National University Yangsan Hospital Institutional Review Board and the Ulsan National Institute of Science and Technology Institutional Review Board in accordance with the guidelines of the Helsinki Declaration. All methods were carried out in accordance with the approved guidelines.

### Tissue Sample

Postmortem midbrains were obtained from a 40-year-old male subject (subject I) and a 70-year-old female subject (subject II), neither with a history of neurological disease. Each subject joined the Pusan National University Anatomical Donation Program and signed the informed consent. The brain tissue was fixed and preserved in 4% neutral buffered formaldehyde solution for at least 2 months (5 and 3 months, respectively), a time period that facilitates a constant tissue T_2_^18^,^19^. The postmortem interval before fixation was less than 24 hours. Each brain sample was sectioned into 1.5-cm-thick slices containing the rostrocaudal extent of the SN parallel to a plane bisecting the mammillary body and the superior colliculus^4^ and was transected in the midsagittal plane to provide one-half (right side) of the SN for the analysis. The SN was normally pigmented without gross abnormalities. All accessible blood vessels were carefully removed to prevent susceptibility artefacts from intravascular iron^20^.

### MRI

MR images were acquired using a 7T MRI system (Bruker Biospec preclinical scanner, Ettlingen, Germany). In order to avoid susceptibility artefacts at the tissue-air interface, the tissue block was inserted into a syringe tube and immersed in a 4% formaldehyde solution. The tube containing the tissue block was then inserted into a magnetic resonance coil (radiofrequency transceiver volume coil, diameter 40 mm). All sequences were acquired along a transverse slice plane parallel to the block face of the rostral SN. To obtain optimal SN contrast, MR images were acquired with the following parameters. A multi-gradient echo sequence was performed to obtain T_2_*WI and T_2_* maps with repetition time (TR) = 2000 ms and echo time (TE) = 3.1~40 ms (10 TEs, increment = 4.1 ms), FOV = 35 × 35 mm, matrix size = 256 × 256, in-plane resolution = 136 × 136 μm, slice thickness = 0.5 mm, number of slices = 20, flip angle = 30°, scanning time = 25 min. Corresponding T_2_* maps were generated by linear fitting from a semi-log plot of decaying signal versus TE using MATLAB (R2013a, The MathWorks, USA). T_1_WI was acquired using a fast spin echo sequence (TR/TE = 700/8.0 ms, FOV = 35 × 35 mm, matrix size = 256 × 256, in-plane resolution = 136 × 136 μm, slice thickness = 0.5 mm, number of slices = 20, flip angle = 90°, scanning time = 96 min). A magnetization transfer contrast pulse (flip angle = 117°, 1500 Hz off-resonance, scanning time = 96 min) was also applied to obtain NM-sensitive T_1_WI.

### Histology

After MRI scanning, the tissue blocks were taken out of the tube and immersed in a 4% formaldehyde solution. Tissue blocks were placed in a solution of 30% sucrose in phosphate-buffered (PB) saline for several days until they sank to the bottom of the vessel. They were then removed from the sucrose bath, frozen in powdered dry ice, and stored at −80 °C. The tissue block was trimmed and sectioned at a thickness of 50 *μ*m parallel to the block face. The histological sections were stored in individual wells in a PB solution containing 0.1% sodium azide at 4 °C. Of ten serial histological slides corresponding to each MRI slice (thickness 0.5 mm), four adjacent slides were stained serially with Perl’s Prussian blue (which is sensitive to ferric iron) without a counter stain, Kluver-Barrera (KB) (luxol fast blue stain for myelin with Nissl counterstain for neurons), tyrosine hydroxylase (TH) (to identify dopamine cells and fibres), and calbindin D_28K_ (to subdivide the SN), using methods previously described^1^,^21^. The sections for Perl’s iron staining were selected to visualize NM, which appear as unstained brown pigments^11^.

Perl’s reaction was performed in a 1:1 mixture of 20% potassium ferrocyanide and HCL. For KB staining, sections were incubated in 0.1% luxol fast blue MBS solution and counterstained in 0.1% cresyl violet acetate solution. For the TH immunohistochemical staining, the sections were incubated in the primary antibody (rabbit anti-tyrosine hydroxylase, AB152, Millipore Corporation, Temecula, CA) at a dilution of 1:500 in 1% normal goat serum, followed by biotinylated goat anti-rabbit immunoglobulin G (1:200 dilution). Immunohistochemical staining for calbindin D_28K_ was performed using the rabbit anti-Calbindin D-28K (1:500 dilution, AB1778, Millipore Corporation, Temecula, CA) and biotinylated goat anti-rabbit immunoglobulin G (1:200 dilution). All slides were scanned using Olympus Slide virtual microscopy (Olympus, Japan) with a spatial resolution of 0.6836 *μ*m^2^ per pixel (100×) and stored in lossless compression formats (Olympus virtual slide image format.vsi and JPEG 2000.jpx).

### Matching between MRI and histology

Careful consideration was required to find accurate corresponding histological slides for each MRI slice. Since the block face of the tissue was used to determine both the direction of MRI acquisition and the histology slide cutting, this information was used to simplify a transformation model between 3D MRI volume and a stack of 2D histology images. We modelled the *z*-direction transformation (perpendicular to the block face) as a global translation and the *xy*-plane transformation (parallel to the block face) as a slice-by-slice 2D rigid transformation to account for possible misalignment during staining and scanning. Based on this transformation model, an up-sampled 3D volume of T_1_WI (68.36 *μ*m^2^ per pixel) was then registered to a stack of down-sampled 2D KB staining images (68.36 *μ*m^2^ per pixel) to maximize mutual information (MI)^22^. In Supplementary Figure S1, the original T_1_WI (C-I) was registered to the KB stain image (A) to obtain the aligned T_1_WI (C-II). Up-sampling was done with a cubic B-spline interpolation and a non-negativity constraint^23^; this image registration code with the above transformation model was implemented using the MATLAB Image Processing Toolbox (The Mathworks, USA). The resulting registration information was used to align other MRI images (e.g., T_2_*WI) to the corresponding KB staining images since all MRI images with different contrasts were obtained without moving the target tissue. In Supplementary Figure S1, the aligned T_2_*WI (D-II) was obtained by registering the original T_2_*WI (D-I) to the KB stain image (A) using the same transformation information obtained from the T_1_WI to KB image registration. Lastly, TH, Perl, and calbindin staining images (10×, 6.836 *μ*m^2^ per pixel) were registered to the closest KB staining image (10×, 6.836 *μ*m^2^ per pixel) using a MI-based rigid image registration. Since image features of each stained image are quite different from one another, we used thresholded binary stained images of KB, TH, Perl, and calbindin to perform image alignments so that no strong internal correlation between images would affect the results of image alignment. In Supplementary Figure S1, the aligned binary Perl stained image (B-II) was obtained from the original Perl stained image (B) that was automatically thresholded to generate the binary image (B-I) using red channel information, which has the largest contrast between the Prussian blue-stained tissue and the white slide background. It was then registered to the binary image (A-I) generated from the KB stained image (A) using red channel information, which also has the highest contrast between the KB blue-stained tissue and the white background. Similarly, the aligned Perl stained image (B-III) was made using the same transformation, in which all three channels of information from the original Perl stained image (B) was aligned to the original KB stained image (A). For the TH and calbindin stained images, similar procedures were applied so that the registered stained images to the KB stained image were obtained. We visually confirmed that our co-registration method between the various MR images and histology images worked well for small tissues cases, like the SN. However, more sophisticated methods such as the work of Adler *et al.*^24^ may be required for imaging larger tissue samples.

### Quantitative analysis between aligned MRI and histology images

Neuron-occupied areas (Supplementary Figure S2, A-II and B-II) were extracted from 2D KB and TH stained images (Supplementary Figure S2, A-I and B-I, respectively, 100×, 0.6836 *μ*m^2^ per pixel) using black and brown colour information for the quantification of Nissl-positive neurons and TH-positive neurons, respectively. Black colour information in blue KB stained sections was extracted by thresholding blue channel information and dark brown colour information in TH-stained sections was extracted by manually selecting the brown-colour-range of the synthetic image from all three colour channels (red channel × max image intensity^2^ + green channel × max image intensity + blue channel). The number of pixels occupied by neurons for each 10 × 10 pixel block was then recorded to generate a density map (% occupied by neurons), as shown in Supplementary Figure S2, D-II (TH case only). Similarly, iron pigments (Supplementary Figure S2, C-III) and unstained pigmented NM (Supplementary Figure S2, C-II) were also extracted from 2D Perl-stained images (Supplementary Figure S2, C-I, the same two figures) to obtain iron and NM density maps (blue and brown, respectively). Blue-coloured iron information was extracted by thresholding the synthetic image that emphasizes the blue colour (blue channel – red channel/2 – green channel/2) and brown-coloured NM information was obtained by thresholding the sum image of all colour channels to distinguish brown-coloured pigments from the white-coloured background. These density images (10×, 6.836 *μ*m^2^ per pixel) were then registered to the closest KB-stained image (10×, 6.836 *μ*m^2^ per pixel) using the previously obtained alignment information. In Supplementary Figure S2, the TH-positive neuron density image (D-II), ranging from 0 to 100%, was registered to the closest KB-stained image to yield the aligned density image (E-II) using the same warp transformation information from the original TH-stained image (D-I) to the aligned image (E-I). Next, all registered MR images and density map (neuron, NM, and iron) images (10×, 6.836 *μ*m^2^ per pixel) were down-sampled to smaller images after twice blurring using a 10 × 10 moving average kernel so that low resolution images (683.6 *μ*m^2^ per pixel) were obtained for a voxel-wise statistical analysis. Histograms of the T_2_* distribution were generated for the respective thresholded binary stained images of iron pigments (blue), NM (brown), and reference tissue regions from the Perl-stained images (10×, 6.836 *μ*m^2^ per pixel). Mean and standard deviation of each T_2_* distribution was obtained and compared to one other.

Voxel-wise correlation analyses were performed between the MR images and the density maps derived from the histology images. No further comparisons between MR and histology images were performed when tissue damage or MRI boundary image artefacts were visually distinct or when the correlation values of neuronal density between KB, TH, and Perl stains were low from possible co-registration error. With these criteria, six sequential slides from each subject were studied. These included the caudal to rostral areas of the SN. Three different polygonal regions of interest (ROIs) were manually selected (Supplementary Figure S3): ROI-whole SN contains the whole SN based on KB staining corresponding to the bulk of SN hypointensity on the T_2_*WI; ROI-SNc contains the SNc based on TH staining within the ROI-whole SN including the A9 cell group, and ROI-SNr contains the SNr, which is obtained by subtracting ROI-SNc from ROI-whole SN. For voxels within ROIs, direct correlations between the T_2_* values and the density of neurons, NM, and iron pigments were evaluated.

Pearson’s linear correlation (Spearman’s rank correlation in parentheses) coefficient tables (5 × 5 matrix each) were constructed for histological variables, the Nissl-positive neuron density, TH-positive neuron density, NM density, and iron pigment density, for the ROI-whole SN using the MATLAB Statistics Toolbox (The Mathworks Inc., USA) for each subject. Pearson’s linear partial correlation (Spearman’s rank partial correlation in parentheses) coefficients were also obtained to measure the degree of association between T_2_* values and each single histological variable with the effects of the other histological variables removed (NM with the effect of iron pigments removed in ROI-SNc, iron pigments with the effect of NM removed in ROI-whole SN, ROI-SNc, and ROI-SNr) using the MATLAB Statistics Toolbox. The coefficients of multiple correlations were calculated for each subject to study the association between the T_2_* values and all histological variables from the above correlation coefficient tables. From our multiple correlations study, we found that more than one variable, including the Nissl-positive neuron density, TH-positive neuron density, and NM density did not improve the coefficient of multiple correlations due to very high correlations among these three variables (see Supplementary Table S1). Therefore, only three variables (T_2_* value, NM density, and iron pigment density) were used for this study. Furthermore, to measure the independent contribution of iron and NM in T_2_* contrast, multiple regression β values for each subject were calculated with y = β_0_ + β_1_x_1_ + β_2_x_2_, where y, x_1_, and x_2_ correspond to is T_2_* values, NM, and iron density values, respectively.

## Results

### Postmortem 7T MRI: the delineation and inner organization of the SN

The *ex vivo* SN imaging protocol was optimized for high resolution histological comparisons and enhanced SN-associated MRI contrasts. For the T_2_*WI, an echo time of TE = 15.4 ms yielded the best MRI visual contrast among the 10 different TEs, ranging from 3.1 to 40 ms (Supplementary Figure S4). All MRI scans displayed visible contrasts between the SN and surrounding structures. The T_1_WI images presented more distinct boundaries than the T_2_* weighted sequences, which appeared as arch shapes between the SN and the crus cerebri. However, the magnetization transfer T_1_WI could not visually depict NM-related contrasts in the SNc as previously reported with 3T MRI^2^,^3^,^7^,^8^,^9^.

**Figure 1:**
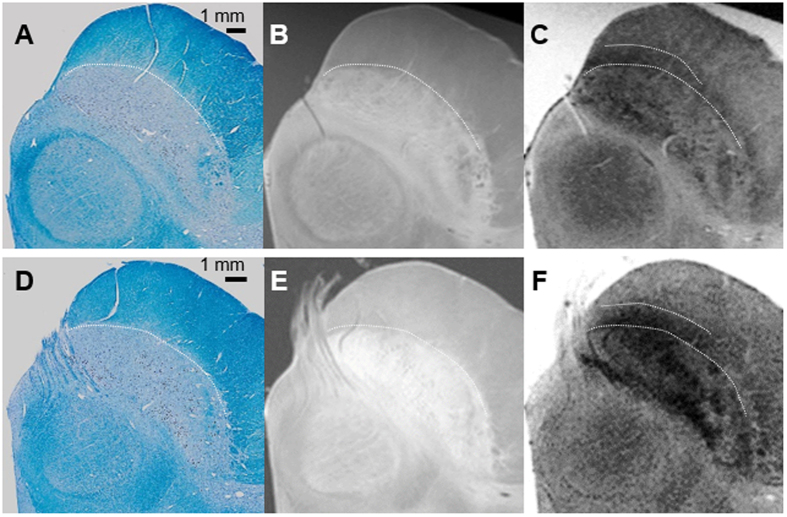
The line of demarcation between the substantia nigra and the crus cerebri (**A**–**C**): the 40-year-old male subject; (**D**–**F**): the 70-year-old female subject). T_1_-weighted hyperintensity corresponds exactly to the extent of the substantia nigra delineated by myelin staining (luxol fast blue) without overlapping with the adjacent crus cerebri. The ventral hypointense layers visible in T_2_*-weighted images extended anteriorly to the posterior part of the crus cerebri.

In both subjects, the ventral hypointense layers visible in the T_2_*WI images extended anteriorly to the crus cerebri (Fig. 1). This signal hypointensity was more prominent in the medial aspect. The boundaries between the SNr and the SNc were difficult to draw regardless of the imaging sequence. We could not find a signal-intensity difference leading to a delineation of these areas. The lateral SN showed hyperintensity relative to the medial areas in the T_2_*WI sequence.

**Figure 2:**
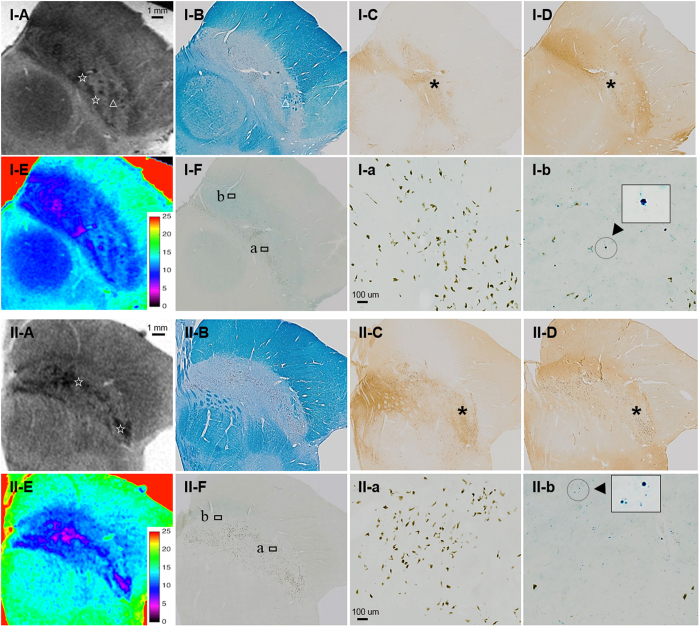
MRI and histology data from the middle of the substantia nigra (SN). The clusters of neuromelanin (NM)-containing dopaminergic neurons were revealed as T_2_* hypointensity (☆) and low T_2_* values within the dorsolateral were revealed as hyperintense areas with a high tyrosine hydroxylase and low calbindin content (*). The regions of high NM content (a) were mostly distinct from those with a high iron content (b) as detected by Perls’ stain (Arrowhead: iron pigments). The clusters of NM exhibited better contrast in the older, 70-year-old female subject (II). Bundles of myelinated fibres in the ventrolateral SN also appear hypointense on T_2_*WI (Δ). A: T_2_*weighted imaging, B: Kluver-Barrera, C: Tyrosine hydroxylase immunohistochemistry, D: calbindin immunohistochemistry, E: T_2_* map (colour bar: T_2_* values), F: Perls’ Prussian blue.

The T_2_*WI showed clusters of hypointense foci within the SN (Figs 1 and 2). Various shapes, including patches or linear streaks, were commonly identified at the exit level of the third cranial nerve fibres. These foci were much more hypointense and had lower T_2_* values than those seen in the hypointense layers between the SN and the crus cerebri.

### Histological components contribute to the MRI contrast

The line of demarcation between the SN and the crus cerebri (Fig. 1) was determined by KB staining. We found that hyperintense areas in T_1_WI corresponded exactly to the extent of the SN, as delineated by histology. However, the ventral hypointense layers on the T_2_*WI were not coextensive with the SN, but instead extended partially into the posterior part of the crus cerebri and overlapped with the densely myelinated portions. In these areas, iron pigments stained using Perl’s technique were detected in the absence of TH-positive cells or NM (Fig. 2).

Co-registration of MRI and histology data allowed us to identify clusters of pigmented NM revealed as prominent T_2_* hypointense in the background of the relative isointense signal (Fig. 2-C,D). Furthermore, MRI contrast in the dorsolateral SN revealed that high TH and low calbindin content was primarily generated by NM-pigmented neurons rather than Perl-positive iron particles (Fig. 2-F,a,b). Some hypointense areas reflected bundles of myelinated fibres in the ventrolateral SN (Fig. 2-A). Among midbrain dopaminergic cell groups, the T_2_* hypointense foci corresponded well to the clusters of NM-containing A9 nigral cell groups (Fig. 2). Similar signal loss was not prominently observed in the other dopaminergic cell groups located in the ventral tegmental (A10) or retrorubral (A8) areas, where there was a lesser amount of NM. The clusters of NM-containing neurons exhibited qualitatively better contrast in the images of the older subject (Fig. 2-II).

**Figure 3:**
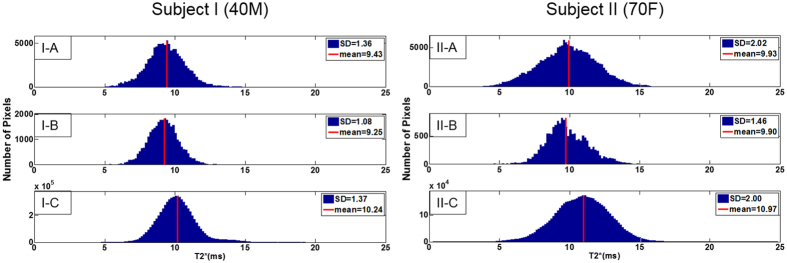
Histogram of T_2_* distribution of thresholded binary stained images of iron and neuromelanin (NM) pigments, and reference region from the Perl stain image substantia nigra (SN). Significantly reduced (*P* < 0.0001) mean T_2_* values were observed for both iron (blue) pigments and NM (brown) with respect to the reference SN tissue region as shown in T_2_* histogram for both the 40-year-old male (subject I) and 70-year-old female (subject II) subjects. A: NM, B: iron pigments, C: reference SN tissue region.

The histological assessment showed that Nissl-positive or TH-positive neurons within the SN were colocalized with pigmented NM. The densities of these histological components were positively correlated with one other but not necessarily with the density of the iron pigment (Supplementary Table S2). Significantly reduced (*P* < 0.0001) mean T_2_* values were observed for both iron pigments and NM with respect to the reference SN tissue region as shown in the T_2_* histogram for both subjects (Fig. 3). Iron pigments and NM showed similar mean T_2_* values. We observed that the T_2_* values were well correlated with NM in the ROI-SNc (Table 1). The Pearson’s partial correlation coefficient was higher for subject II (*r* = −0.47 for subject I, *P* < 0.0001 and *r* = −0.65 for subject II, *P* < 0.0001). T_2_* values were also significantly correlated with iron pigments within the ROI-whole SN, ROI-SNc, and ROI-SNr in both subjects (Table 1; *P* < 0.0001). Note that both Pearson’s linear correlations and nonparametric Spearman’s rank correlations yielded similar results. Coefficients of the multiple correlation (*R*) between T_2_* values, iron pigments, and NM were 0.56 and 0.70 for subject I and subject II, respectively. When β_1_ is for NM and β_2_ is for iron in each subject, respectively, subject I was found to have β_0_ = 10.83, β_1_ = −0.43, and β_2_ = −8.05 while subject II had β_0_ = 11.95, β_1_ = −0.83, and β_2_ = −12.64. Both cases had higher β-values for iron than NM. Considering that β_1_ indicates the contribution of NM to T_2_* and β_2_ is the contribution of iron to T_2_*, the relative contribution of iron to T_2_* is higher than the contribution of NM to T_2_* by a factor of 18.7 and 15.2 times for subjects I and II, respectively. In other words, one particle of iron confers about −10 (ms) to the T_2_* value of corresponding region, while 15 particles of NM confer about −10 (ms) to the T_2_* value of the corresponding region. However, mean T_2_* values in Fig. 3 were similar in both iron and NM pigments since the number of NM particles was much larger than that of iron particles from Perl-stained slides.

## Discussion

We identified one of the main histological components that contribute to T_2_*-sensitive contrast by using the co-registration of postmortem MRI and histological data. Signal loss on T_2_*WI may be attributable to paramagnetic macromolecules, both NM and iron pigments, within the SN^4^. These quantitative associations suggest that NM may be one of the determinants of T_2_* signal loss in the SNc.

Correlations between the MRI data and histology is required to elucidate the nature of the MRI contrast. Histologically validated high-resolution MRI may enable a more precise definition of the boundaries and substructures of the SN. In previous studies, the overall correlation was calculated either based on a visual comparison between MRI and histological sections or with atlases obtained from different subjects^25^. However, visual comparison is prone to misalignment error. Recently, more precise postmortem high-resolution MRI and histological correlations from the same sections were performed using semi-automatic co-registration methods based on manually placed landmarks or block-face images^11^,^21^. In this study, we used a fully automated MI-based image registration method to align the MRI and histology images with a new transformation model (2D rigid transformation + 1D translation) rather than a fully 3D rigid transformation model, to account for possible transformations that happen during slice preparation. In addition, our image registration used thresholded binary histology images such that a localized registration-induced bias would not be introduced in the correlation studies between T_2_*-related MR images and histology images (except for the T_1_WI to KB-stained image registration).

The distribution of iron in the SN has been localized to NM-containing neurons, microglia, and astrocytes. NM is the principal iron storage site and plays an important role in intraneuronal iron homeostasis in dopaminergic neurons of the SN^26^. By visual comparison, we found that the paramagnetic hypointense foci with decreased T_2_* values within the dorsolateral SN corresponded well to the clusters of NM-containing neurons, although not accompanied by sufficient amounts of stainable iron pigments.

It is likely that NM-bound iron causes paramagnetic T_2_ shortening effects^4^. Our results also suggest that NM is one of main sources of a T_2_*-sensitive contrast in the SN at 7T. The correlation of T_2_* shortening with the density of NM and the negligible T_2_* changes in the less-pigmented dopaminergic neurons of the ventral tegmental (A10) and retrorubral (A8) areas support this hypothesis^20^. However, we are unable to provide histological evidence of the amount of iron bound to NM leading to T_2_* contrast because ferric iron deposits in NM-containing neurons are not usually detectable by Perls’ staining^15^.

We observed some individual differences in the contribution of NM and iron to T_2_* signal loss. The multiple correlation analysis showed that the contribution of NM and iron to T_2_* signal loss was greater in the older subject. This may be explained by postmortem evidence for age-related increases in these two histological variables^3^,^26^,^27^,^28^. The content of NM-iron complexes is known to increase with age^15^,^26^,^27^,^28^. NM deposits in the extracellular space were also observed in the more aged SN^15^,^26^. Indeed, the presence of significant amounts of NM released from dying neurons is a feature of PD^15^,^26^. Unfortunately, the staining techniques used in this study were not able to differentiate between intraneuronal and extraneuronal NM. Future investigations will be pursued to further explore these results with a larger, age-controlled sample.

Besides macromolecules (neurons, pigmented NM) and iron pigments, other local tissue components can influence T_2_* contrast, such as water content changes, microvasculature, as well as the tissue properties of the postmortem brain^29^. We found hypointense areas indicating bundles of myelinated fibres, particularly in the ventrolateral SN. Previous *in vivo* studies have shown vascular contrasts corresponding to Duvernoy’s India ink-stained atlas images. They suggested that the non-heme iron visualized in T_2_*WI or SWI are most likely due to the ferritin associated with the vascular network^4^,^30^. In contrast to this hypothesis, we could not find clear vessel-related contrast within the postmortem SN. Although its feasibility is questionable, further correlative analyses of *in vivo* MR images with postmortem histological sections from the same subject are needed to confirm the presence of vascular signals.

Recent studies have shown that the line of demarcation between the SN and the crus cerebri in T_2_ imaging is ambiguous^25^. We observed iron-related signals with a band-like appearance in the SNr, which extended anteriorly to the posterior crus cerebri. Histologically, the hypointense areas on the T_2_*WI overlapped with densely myelinated fibres delineated by KB staining. Additionally, the margins of the SN facing the crus cerebri were very well correlated with the calbindin-stained fibres, which coincides with the striato-pallido-nigral pathway^3^,^31^. At the cellular level, the Perl staining for iron showed abundant iron pigmentation, presumably in the oligodendrocytes, which are known to stain more strongly for iron than any other cell type^3^,^4^. Therefore, it is reasonable to infer that the hypointense area posterior to the crus cerebri is the transitional part of the SNr. The T_1_WI hyperintensity located in the same area corresponded exactly to the extent of the SN as delineated by myelin-stained histology without overlapping with the adjacent crus cerebri. This is likely due to the fact that the MR T_1_ shortening effect from iron is weaker and more localized than those from blurring the T_2_* effect in this area^4^, especially at a high field strength. Recent studies also indicate that the boundaries of the SN derived from the NM-sensitive MRI and T_2_WI/SWI were spatially incongruent^21^,^29^. This discrepancy between different MRI contrasts should be considered in interpreting the resultant delineations of the SN^29^.

As with any postmortem study, the effects of tissue fixation must be considered^19^. The MRI properties of postmortem tissue can change as a result of decomposition and chemical fixation. T_2_ values have been observed to be lower in fixed postmortem brain than *in vivo* for both white and grey matter^18^. The postmortem brain tissue samples in this study were fixed for at least two months, as required for T_2_ values to stabilize^18^,^19^. The overall distribution of signal abnormalities are similar to those obtained in previous postmortem MRIs, which showed more laminar structure in the SN compared to *in vivo* scans^6^,^11^. T_1_-weighted sequencing with the magnetization transfer contrast pulse used here did not create an NM-specific contrast in the SNc^10^,^11^. The reduction of T_1_ contrast at the high field strength and the proton microenvironment may have limited tissue T_1_ contrast due to changes in the penetration of the tissue by the fixative^20^,^32^.

We identified the NM-related T_2_* hypointense signal with the co-registration of 7T MRI and histology that accounts for the steps in histology slice preparation. Metal-bound NM macromolecules can alter the magnetic field uniformity and cause paramagnetic hypointense signals in T_2_*WI and decreased T_2_* values within the SN^4^. Nigral T_2_* imaging can reflect the cellular density of NM-containing neurons at 7T. Future investigations with increased sample sizes and advanced MRI techniques are necessary to generalize our observations, including *in vivo* verifications^6^,^11^.

## Additional Information

**How to cite this article**: Lee, J.H. *et al.* The Neuromelanin-related T_2_* Contrast in Postmortem Human Substantia Nigra with 7T MRI. *Sci. Rep.* **6**, 32647; doi: 10.1038/srep32647 (2016).

## Supplementary Material

Supplementary Information

## Figures and Tables

**Table 1:** Pearson’s partial correlations (Spearman’s partial correlations in parentheses) between the T_2_* values and the histologic densities in the 40-year-old male subject (subject I) and the 70-year-old female subject (subject II).

Pearson’s Partial Correlations(Spearman’s Partial Correlations)	Subject I	Subject II
Neuromelanin		
ROI-SNc	−0.47* (−0.42*)	−0.65* (−0.61*)
Iron		
ROI-whole SN	−0.48* (−0.60*)	−0.53* (−0.63*)
ROI-SNc	−0.56* (−0.60*)	−0.45* (−0.54*)
ROI-SNr	−0.46* (−0.60*)	−0.63* (−0.78*)

Neuromelanin and iron densities were extracted from a single histologic slice for the corresponding MRI slice. Six histologic and MRI slices were used in the calculations.

ROI: region of interest, SN: substantia nigra, SNc: the substantia nigra pars compacta, SNr: the substantia nigra pars reticulata. *P < 0.0001.
