# Supplementary material for: The Neuromelanin-related T2* Contrast in Postmortem Human Substantia Nigra with 7T MRI
Source: Sci Rep. 2016 Sep 6;6:32647. doi: 10.1038/srep32647 (PMC5011647; doi:10.1038/srep32647)
Supplement: Supplementary Information [file srep32647-s1.pdf]

Supplementary Information

The Neuromelanin-related T_2* Contrast in Postmortem Human Substantia Nigra with 7T MRI

Jae-Hyeok Lee [a,1], Sun-Yong Baek [b,1], YoungKyu Song [c], Sujeong Lim [c], Hansol Lee [c], Minh Phuong Nguyen [d], Eun-Joo Kim [e], Gi Yeong Huh [f], Se Young Chun [d,*], HyungJoon Cho [c,*]

[a] Department of Neurology, Research Institute for Convergence of Biomedical Science and Technology, Pusan National University Yangsan Hospital, Yangsan, South Korea

[b] Department of Anatomy, Pusan National University School of Medicine, Yangsan, South Korea

[c] Department of Biomedical Engineering, Ulsan National Institute of Science and Technology, Ulsan, South Korea

[d] School of Electrical and Computer Engineering, Ulsan National Institute of Science and Technology, Ulsan, South Korea

[e] Department of Neurology, Pusan National University Hospital, Busan, South Korea

[f] Department of Forensic Medicine, Pusan National University School of Medicine, Yangsan, South Korea

[1] Co-first authors.

[*] Corresponding authors.

E-mail addresses: sychun@unist.ac.kr (S.Y.C.), hjcho@unist.ac.kr (H.J.C.).

Supplementary Figure S1

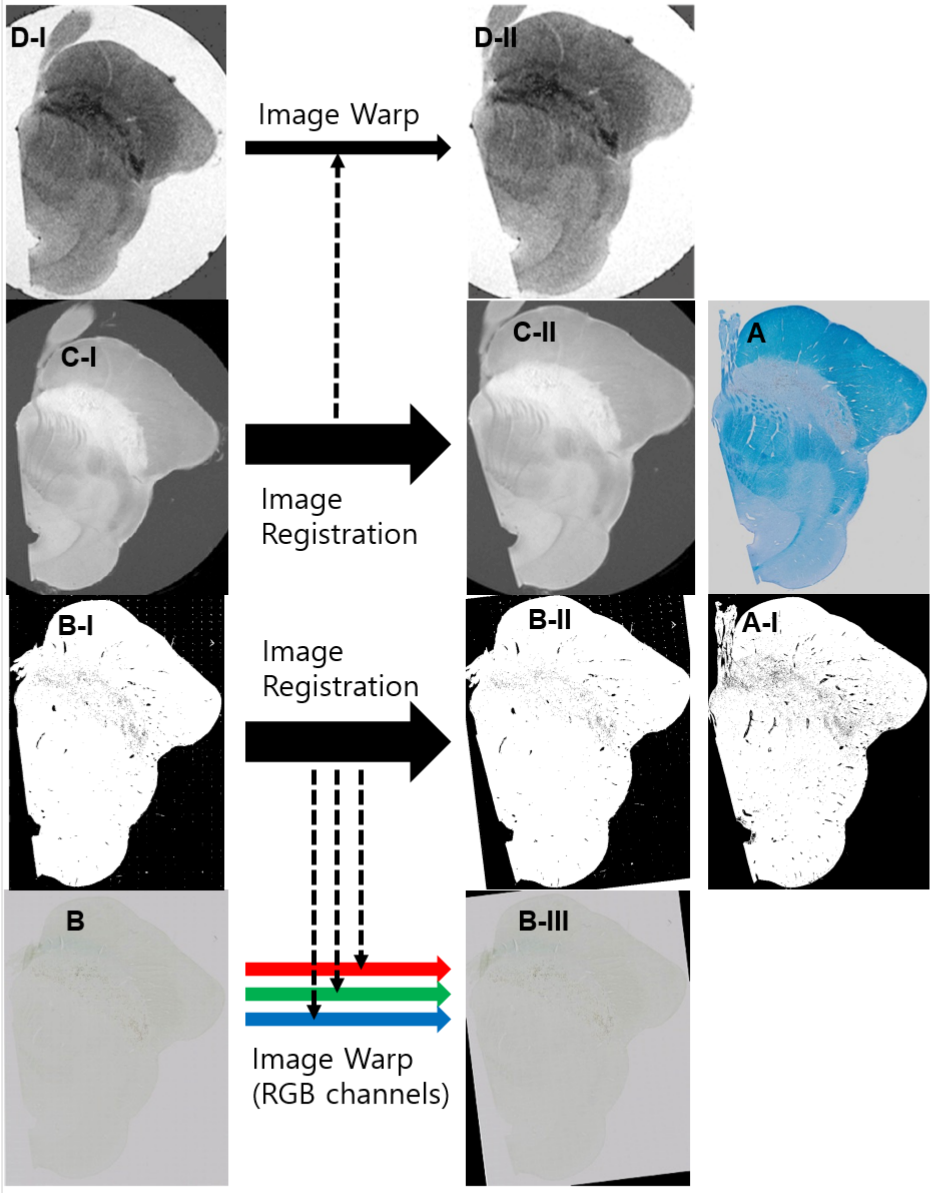

Supplementary Figure S1. The schematic diagram of the histology-MRI image registration. The binary Perl image (B-I) (from the Perl stain image (B)) was registered to the binary KB image (A-I) (from the KB stain image (A)) to yield an aligned binary Perl image (B-II). Then, the resulting transformation information was used to warp the RGB channel information from the original Perl stain image (B) to generate the aligned Perl stain image (B-III). The original T_1WI (C-I) was registered to the KB stain image (A) using red channel information, to yield the aligned T_1WI (C-II). The same transformation information was used to warp the original T_2*WI (D-I) to the registered T_2*WI (D-II).

Supplementary Figure S2

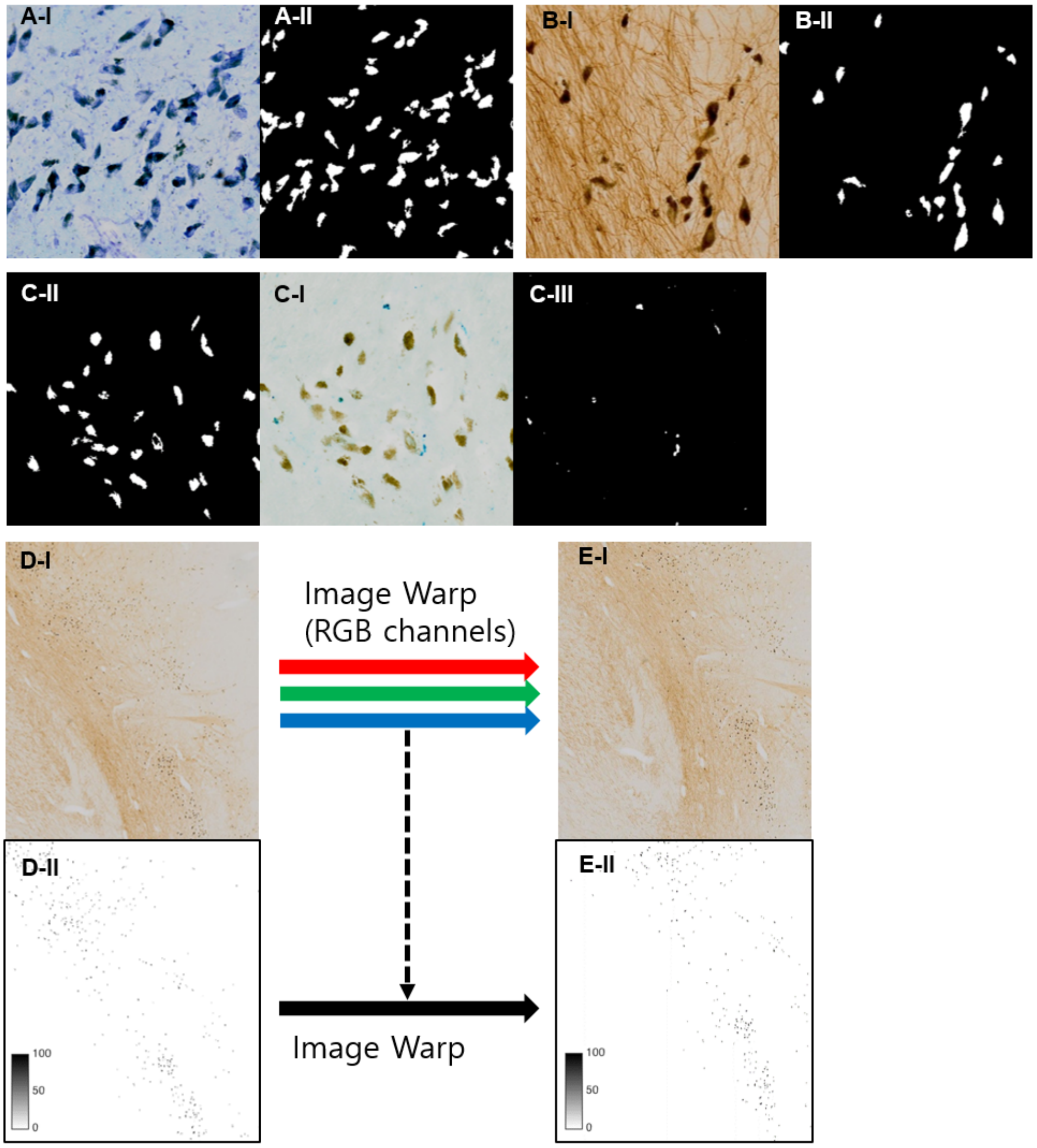

Supplementary Figure S2. Neuron-occupied areas (A-II and B-II) were extracted from 2D KB (A-I) and TH (B-I) stained images. Iron pigments (C-III) and unstained pigmented NM (C-II) were also extracted from 2D Perl stained images (C-I, the same two figures) to obtain iron and NM density maps. Then, the number of pixels occupied by neurons or pigments per 10×10 block was recorded to generate a density map (% occupied by neurons) as shown in D-II (only TH case shown). Then, the TH-positive neuron density image (D-II) ranging from 0 to 100% was registered to the closest KB stain image to yield the aligned density image (E-II) using the same transformation information to warp from the original TH stain image (D-I) to the aligned image (E-I).

Supplementary Figure S3

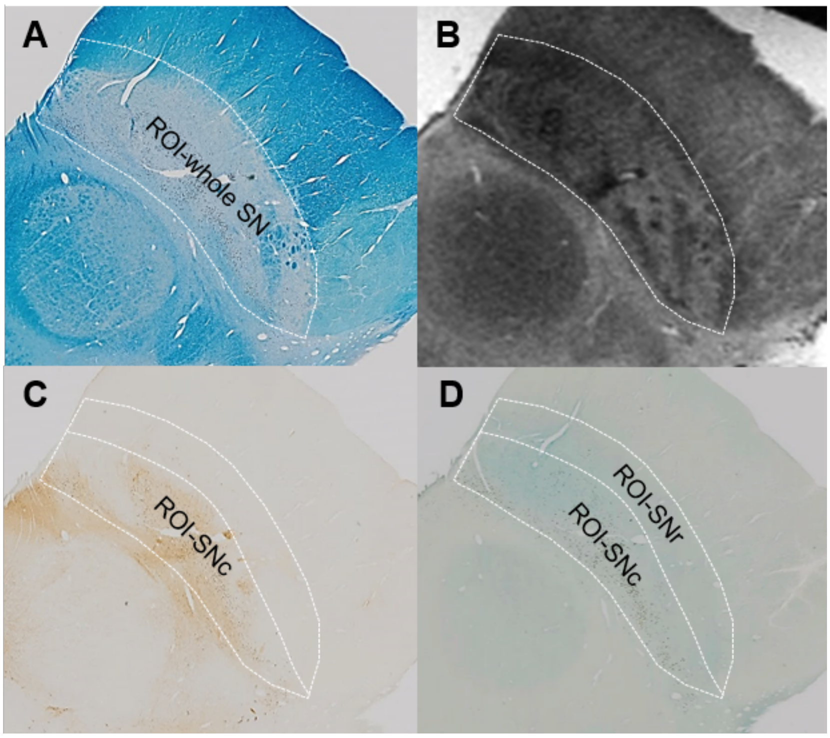

Supplementary Figure S3. The polygonal regions of interest (ROIs). ROI-whole SN (A) based on the KB stain corresponding to the most of SN hypointensity on the T_2*WI (B), ROI-SNc (the substantia nigra pars compacta) based on the TH stain within the ROI-whole SN including the A9 cell group (C), and ROI-SNr (the substantia nigra pars reticulata) obtained by subtracting ROI-SNc from ROI-whole SN (D).

Supplementary Figure S4

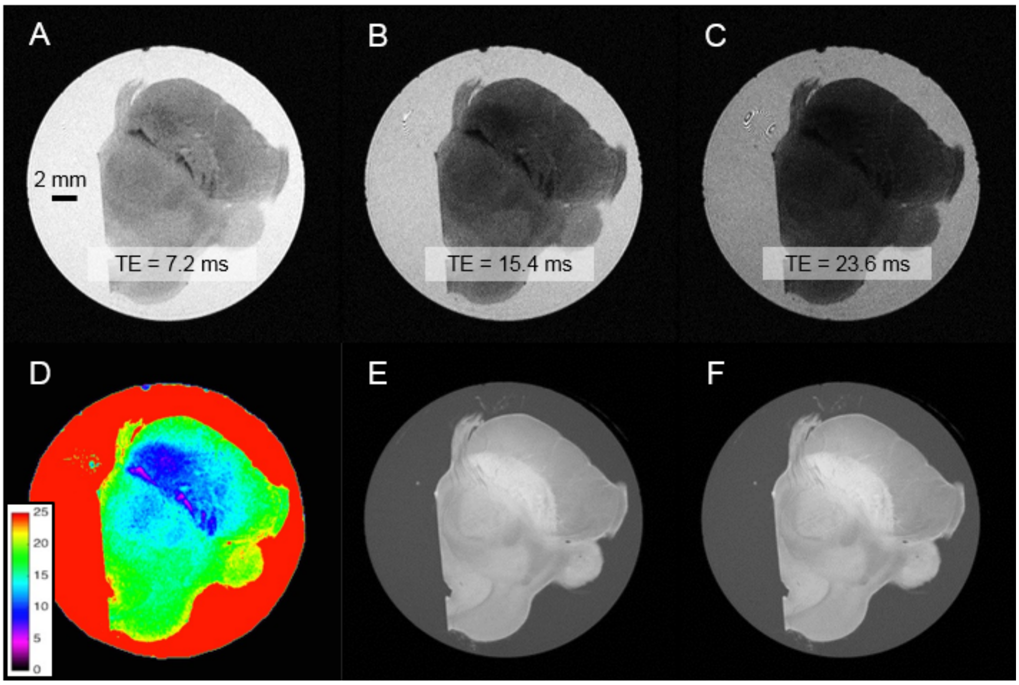

Supplementary Figure S4. The ex vivo substantia nigra MRI protocol (70-year-old female subject, at the rostral level the exiting third cranial nerve fibers). For the T_2*WI, an echo time of TE = 15.4 ms yielded the best visual MRI contrast among the ten different TEs, ranging from 3.1 to 40 ms (A, B, C). For the color-coded T_2* map (D), the color bar represents T_2* values (ms). The T_1WI (E) presents arch-shaped boundaries between the SN and the crus cerebri more distinctly than T_2*WI. The magnetization transfer T_1-weighted image (F) is unable to accurately depict NM-related contrasts in the substantia nigra pars compacta.

Supplementary Table S1

Multiple R	Subject I	Subject II
T_2* with Iron	0.49	0.31
T_2* with Iron, NM	0.56	0.70
T_2* with Iron, NM, Nissl	0.56	0.70
T_2* with Iron, NM, Nissl, TH	0.58	0.71

Supplementary Table S1. Coefficients of multiple correlation (multiple R) for T_2* with histologic measures such as iron, NM, Nissl, and TH. These show that Nissl and TH are almost co-linear with NM and Iron since adding Nissl and TH to linear models did not increase multiple R much.

Supplementary Table S2

Pearson's Correlation (Spearman's Correlation)	Nissl	TH	NM
Subject I (six slices)			
TH	0.59* (0.68*)		
NM	0.82* (0.82*)	0.62* (0.66*)	
Iron	0.07 (0.30*)	0.01 (0.19*)	0.05 (0.30*)
Subject II (six slices)			
TH	0.69* (0.77*)		
NM	0.85* (0.86*)	0.69* (0.75*)	
Iron	-0.27* (-0.33*)	-0.17* (-0.12**)	-0.25* (-0.27*)

TH: tyrosine hydroxylase, NM: neuromelanin. * $P < 0.0001$, ** $P < 0.005$

Supplementary Table S2. Pearson's correlations (Spearman's correlations in parentheses) between the densities of the histological components.
